# Durable Anti-Vi IgG and IgA Antibody Responses in 15-Month-Old Children Vaccinated With Typhoid Conjugate Vaccine in Burkina Faso

**DOI:** 10.1093/jpids/piad058

**Published:** 2023-08-17

**Authors:** Alphonse Ouedraogo, Amidou Diarra, Issa Nébié, Nouhoun Barry, Jean Moise Kabore, Alfred B Tiono, Shrimati Datta, Yuanyuan Liang, Ifayet Mayo, Jennifer J Oshinsky, J Kathleen Tracy, Tsion Girmay, Marcela F Pasetti, Leslie P Jamka, Kathleen M Neuzil, Sodiomon B Sirima, Matthew B Laurens

**Affiliations:** Groupe de Recherche Action en Santé, Ouagadougou, Burkina Faso; Groupe de Recherche Action en Santé, Ouagadougou, Burkina Faso; Groupe de Recherche Action en Santé, Ouagadougou, Burkina Faso; Groupe de Recherche Action en Santé, Ouagadougou, Burkina Faso; Groupe de Recherche Action en Santé, Ouagadougou, Burkina Faso; Groupe de Recherche Action en Santé, Ouagadougou, Burkina Faso; Center for Vaccine Development and Global Health, University of Maryland School of Medicine, Baltimore, Maryland, USA; Center for Vaccine Development and Global Health, University of Maryland School of Medicine, Baltimore, Maryland, USA; Department of Epidemiology and Public Health, University of Maryland School of Medicine, Baltimore, Maryland, USA; Center for Vaccine Development and Global Health, University of Maryland School of Medicine, Baltimore, Maryland, USA; Center for Vaccine Development and Global Health, University of Maryland School of Medicine, Baltimore, Maryland, USA; Center for Vaccine Development and Global Health, University of Maryland School of Medicine, Baltimore, Maryland, USA; Center for Vaccine Development and Global Health, University of Maryland School of Medicine, Baltimore, Maryland, USA; Center for Vaccine Development and Global Health, University of Maryland School of Medicine, Baltimore, Maryland, USA; Center for Vaccine Development and Global Health, University of Maryland School of Medicine, Baltimore, Maryland, USA; Center for Vaccine Development and Global Health, University of Maryland School of Medicine, Baltimore, Maryland, USA; Groupe de Recherche Action en Santé, Ouagadougou, Burkina Faso; Center for Vaccine Development and Global Health, University of Maryland School of Medicine, Baltimore, Maryland, USA

**Keywords:** Africa, Burkina Faso, IgA and IgG, immunogenicity, typhoid conjugate vaccine

## Abstract

We assessed anti-Vi IgG/IgA responses to typhoid conjugate vaccine (TCV) in children enrolled in a double-blind randomized controlled, phase 2 trial in Burkina Faso. Anti-Vi IgG seroconversion and anti-Vi IgA titers were higher in TCV than control recipients at 30–35 months post-vaccination. TCV induces durable immunity in Burkinabe children vaccinated at 15 months.

## Teaser

A single dose of typhoid conjugate vaccine (TCV) induces durable immunity in Burkinabe children vaccinated at 15 months 30–35 months post-vaccination. Extended duration data—of a novel TCV in West Africa—will inform policy and decision-makers considering the country introduction of TCV.

## INTRODUCTION

In 2017, the World Health Organization prequalified the first typhoid conjugate vaccine (Typbar TCV) and deems anti-Vi IgG an appropriate measure of vaccine immunogenicity [1]. In adults in the UK, TCV induced high anti-Vi IgG and IgA responses [2, 3]. In addition, anti-Vi IgA antibodies generated by TCV were associated with protection against controlled human infection with *Salmonella* Typhi (*S* Typhi) [3]. Vi IgA and IgG responses together predicted protection from typhoid [4]. In Burkina Faso, TCV was safe and immunogenic when coadministered with measles-rubella (MR) and yellow fever vaccines at 9–11 months [5], and with MR and meningococcal serogroup A conjugate vaccine (MCV-A) at 15–23 months in a double-blind, randomized, controlled Phase 2 clinical trial [6].

We evaluated anti-Vi IgA antibodies 28 days after vaccination, and anti-Vi IgG and IgA antibodies 30–35 months post-vaccination in participants from a clinical trial where TCV was coadministered with routine MR vaccine, with and without MCV-A, at the 15-month vaccination visit [6]. The study was approved by ethics committees in Burkina Faso and Maryland, USA. Parents/guardians of study participants provided informed consent (Clinicaltrials.gov NCT03614533).

## METHODS

Detailed methods of this randomized, double-blinded, controlled, Phase 2 trial were published previously [6]. Briefly, TCV or inactivated polio vaccine (IPV) was administered intramuscularly to children 15–23 months of age. Children were randomized 1:1:1 to Group 1 (TCV and IPV, MCV-A 28 days later), Group 2 (TCV and MCV-A), or Group 3 (MCV-A and IPV). All children received their second dose of MR vaccine. Adverse events were recorded for 7 days (solicited), 28 days (unsolicited), and 180 days (serious) during the clinical trial [6]. Children 15–23 months of age were eligible if their parent/guardian consented. Participants were excluded if they received blood products in the last 6 months and/or had any other condition likely to interfere with immunity or pose a health risk. The extended immunogenicity study protocol is provided in Supplementary Information.

Families were informed about the study and provided written informed consent in a private area before enrollment and venipuncture. Blood was drawn at baseline (Day 0), 28 days, and for participants whose parent/guardian could be recontacted and provided consent for an additional collection, 30–35 months after vaccination. Serum was tested for anti-Vi IgG and IgA antibodies using a commercially available enzyme-linked immunosorbent assay (ELISA) kit, VaccZyme *S* Typhi Vi immunoglobulin IgG (The Binding Site Group Ltd, Birmingham, UK) at the University of Maryland, Baltimore, MD.

### Outcomes

Primary study outcomes were anti-Vi IgA antibody titers at 28 days, and both anti-Vi IgA and IgG antibody titers at 30–35 months post-vaccination. Participants with a fourfold rise in titer from baseline were considered seroconverted.

### Statistical Analysis

Baseline demographics were compared between groups receiving TCV (Groups 1 and 2) and those that received the control vaccine (Group 3), and compared between participants who returned for their 30–35 month visit and participants who did not, using Chi-square test for differences in sex, Mann–Whitney *U* test for age, and two-sample *t*-test with unequal variances for height and weight.

Geometric mean anti-Vi titers were compared between groups using a two-sample *t*-test on log_10_-transformed titer values. According to standard practice when computing geometric means, zeros and values below the detection limit were replaced by one-half the limit of detection. Individual results below the assay detection limit for IgG (7.4 EU/mL) and IgA (3.0 EU/mL) were replaced with one-half this threshold, 3.7 EU/mL and 1.5 EU/mL, respectively. Differences in percent seroconversion were compared using Pearson’s chi-square test or Fisher’s exact test. A *p*-value of ≤0.05 was considered statistically significant. Results were analyzed using SAS software version 9.4 (Copyright 2016; SAS Institute Inc., Cary, NC).

## RESULTS

### Baseline Characteristics

**Figure 1.**
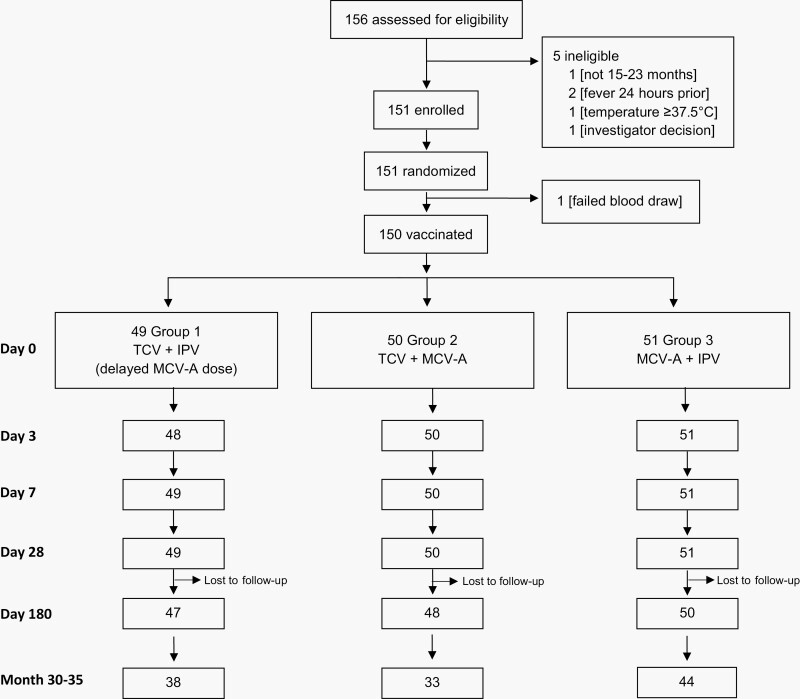
Flow chart showing enrollment, randomization, and loss to follow-up.

Of the 150 participants enrolled and vaccinated, blood was collected at 28 days from 148 children and at 30–35 months from 115 children (Table 1 and Figure 1). Among participants who received TCV (Groups 1 and 2), there were more males and slightly older than participants who received the control vaccine (Group 3), *p* = .03 and .01, respectively. Height and weight were similar between the TCV and control groups, *p* = .66 and .88, respectively. Participants who did not return for their 30–35 month visit had similar baseline characteristics in sex, height, and weight as participants who returned. Those who were lost to follow-up at the 30-35 month visit were slightly older at baseline (Supplementary Table A1).

**Table 1.** Demographics and Anti-Vi IgG and IgA Antibody Responses

	Group 1: TCV + IPV (delayed MCV-A)		Group 2: TCV + MCV-A		Group 3: MCV-A + IPV	
Enrolled^‡^^a^	49		51		51	
Vaccinated	49		50		51	
Sex						
Female	19 (38.8%)		23 (46.0%)		31 (60.8%)	
Male	30 (61.2%)		27 (54.0%)		20 (39.2%)	
Age in months	16.4 ± 1.7		16.1 ± 1.7		15.7 ± 1.2	
Height	75.6 ± 3.9		75.8 ± 3.3		75.4 ± 4.3	
Weight	9.4 ± 1.2		9.5 ± 1.1		9.5 ± 1.2	
Returned at 30–35 months	38		33		44	
Sex						
Female	16 (42.1%)		15 (45.5%)		26 (59.1%)	
Male	22 (57.9%)		18 (54.6%)		18 (40.9%)	
Age in months	47.4 ± 1.6		47.3 ± 1.8		46.6 ± 0.9	
Geometric mean titer	*n*	Mean (95% CI)	*n*	Mean (95% CI)	*n*	Mean (95% CI)
Anti-Vi IgG						
Day 0^b^	49	5.0 (3.9–6.2)	50^c^	4.7 (3.7–5.8)	51	4.8 (3.8–6.2)
Day 28^b^	47^d^	2754.1 (1537.3–4934.1)	50^c^	3707.3 (2632.0–5222.0)	51	5.3 (4.1–6.9)
Months 30–35	38	82.6 (60.0–113.6)	33	75.6 (53.7–106.6)	44	5.7 (4.4–7.3)
Anti-Vi IgA^e^						
Day 28	47^c^	45.5 (33.1–62.4)	50^b^	37.3 (29.3–47.6)	51	1.7 (1.5–1.8)
Months 30–35	38	5.7 (4.0–8.0)	33	4.5 (3.4–6.1)	44	1.7 (1.5–2.0)
Anti-Vi IgG Seroconversion ≥4-fold rise from:	*n*/*N*	% (95% CI)	*n*/*N*	% (95% CI)	*n*/*N*	% (95% CI)
Days 0–28^b^	44/47	93.6 (82.5–98.7)	48/50	96.0 (86.3–99.5)	2/51	3.9 (0.5–13.5)
Day 0 to Months 30–35	34/38	89.5 (75.2–99.2)	29/33	87.9 (71.8–96.6)	3/44	6.8 (1.4–18.7)

Data are *n* (%), mean (±standard deviation) or mean (95% CI). *n* = number of participants. *N* = total number. CI, confidence interval; IPV, inactivated polio vaccine; MCV-A, group A meningococcal conjugate vaccine; TCV, typhoid conjugate vaccine.

^a^Previously published Sirima et al. [6].

^b^Sirima et al. [6].

^c^One participant not vaccinated.

^d^One participant excluded in per-protocol immunogenicity analysis for late Day 28 visit and one participant missing titer, n = 47.

^e^Day 0 anti-Vi IgA not assessed.

### IgG Geometric Mean Titer and Seroconversion at 30–35 Months After Vaccination

**Figure 2.**
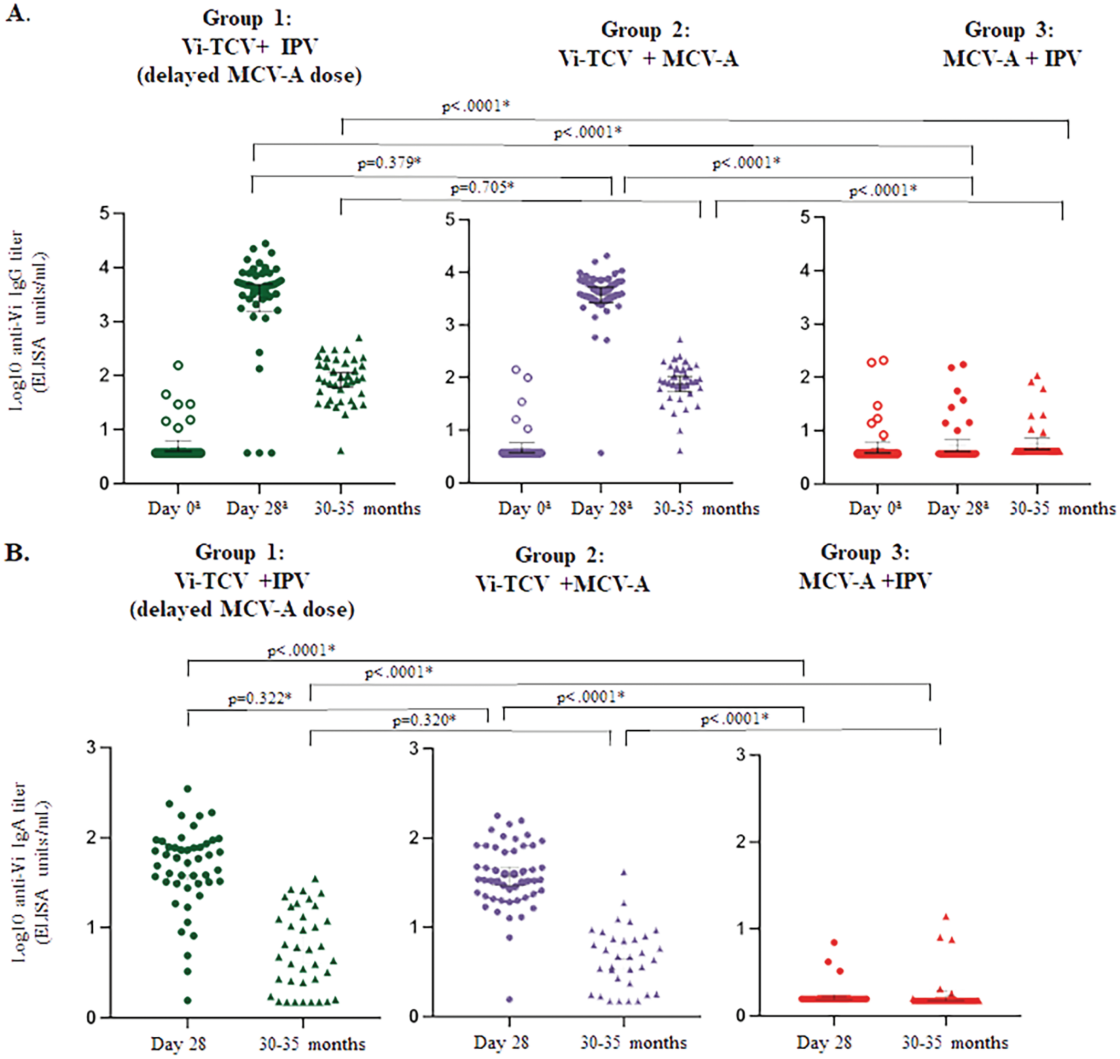
Anti-Vi antibody titers. (A) Anti-Vi IgG antibody titers before vaccination (Day 0), 28 days and 30–35 months after vaccination. (B) Anti-Vi IgA antibody titers 28 days and 30–35 months after vaccination. Circles and triangles represent the log_10_ transformed antibody titer result for each individual participant in each corresponding vaccine group. Bar in the middle of individual participant results for each time point represents the mean antibody titer value on log_10_ scale with 95% confidence interval. *Using two-sample *t*-test with unequal variances on log_10_ transformed data. ^‡^ Previously published, Sirima et al. [6].

Baseline (day 0) anti-Vi IgG antibody geometric mean titer (GMT) was similar for all three groups. As reported previously, titers increased at 28 days after TCV immunization (Table 1) [6]. At 30–35 months post-vaccination, GMT was at least 13-fold greater in TCV recipients versus controls and did not differ between MCV-A or IPV coadministration groups (Figure 2 and Table 1). Anti-Vi IgG seroconversion at 30–35 months post-vaccination was ≥88% in TCV recipients compared to 7% in controls (Table 1). Results did not differ by sex (Supplementary Table A2).

### IgA GMT at 28 Days and 30–35 Months After Vaccination

Day 28 anti-Vi IgA antibody GMT was at least 20-fold higher in children who received TCV versus controls (Table 1). Concomitant versus MCV-A administration 1 month later did not influence Day 28 anti-Vi IgA responses (*p* = .32). By months 30–35, GMT declined for TCV recipients, though remained significantly higher than controls (*p* < .0001; Figure 2). Results did not differ by sex (Supplementary Table A3).

## DISCUSSION

TCV safety, immunogenicity, and efficacy have been demonstrated in adults and children as young as 6 months of age in typhoid-endemic areas [5–10]. A randomized controlled Phase 2 clinical trial in Burkina Faso confirmed TCV safety, noninterference with MCV-A, and robust anti-Vi IgG antibody response 28 days after vaccination [6]. Building on these results, and since no established immunologic correlate of protection exists, we evaluated longer-term IgG and post-vaccination IgA immune responses. With these data, we can infer Burkinabe children vaccinated at 15 months will likely have protection against typhoid until they reach school age, at minimum.

These data demonstrate robust longer-term immunogenicity in children who received coadministered TCV at the 15-month vaccination visit. The presence of comparatively low anti-Vi IgG antibody titers at baseline suggests a lack of environmental exposure to typhoid before 15 months of age and probably the disappearance of pre-existing maternal antibodies that could protect against disease. Increased anti-Vi IgG and anti-Vi IgA antibodies at 30–35 months post-vaccination in TCV recipients supports TCV’s broad and durable immune response for at least 2.5 years. These data support findings from India where GMTs in children 6–23 months vaccinated with TCV were 21-fold, 8-fold, and 5-fold above baseline at 3-, 5-, and 7-years post-vaccination and seroconversion rates were 72% and 44%, at 5- and 7-years post-vaccination, respectively [11]. A study from Malawi also reported favorable safety and durable immunogenicity for up to 3 years after TCV was coadministered with MR vaccine at 9–11 months and when given alone to children up to 12 years of age [12]. TCV was introduced by Liberia, Pakistan, Nepal, Samoa, Zimbabwe, and Malawi. Routine TCV use in these countries will provide additional opportunities to assess TCV immunogenicity in similar settings.

TCV recipients in Burkina Faso, vaccinated at the 15-month immunization visit, reported at least 94% seroconversion 28 days after vaccination in a previous analysis of anti-Vi IgG antibody [6]. Similar robust anti-Vi IgG immune responses were reported in children from Vietnam, Nepal, India, and Malawi [7, 10, 12, 13]. Studies conducted in these countries also document high vaccine efficacy for single-dose TCV, including 80% vaccine efficacy for 18–24 months after vaccination in Malawian children [9]. Our finding of sustained anti-Vi IgG seroconversion of at least 88% at 30–35 months demonstrates the persistence of immunological responses to a single-dose vaccination of TCV and supports that the vaccine is likely protective. Although IgG titers decreased from 28 days to 30–35 months after vaccination, at least 88% of participants remained above the seroconversion threshold. This longer-term seroconversion rate is higher than the 60% documented in India at 25 months [7] and the 80% documented in Malawi at 2–3 years [12], and similar to the 95% documented in Nepal at 18 months [10]. Anti-Vi IgA results from this cohort at 30–35 months are comparable to responses in Nepalese children <5 years of age at 18 months post-vaccination, for whom vaccine efficacy of 79% was demonstrated [10]. Sustained vaccine immunogenicity demonstrated in this study is important because it confirms coadministration of routine MCV-A does not diminish longer-term immune responses to TCV, and that children from distinct geography, climate, and ethnic background maintain a vaccine-induced immune response similar to children from other typhoid-endemic areas that is consistent with protective efficacy against typhoid fever.

In the absence of an ideal assay for functional antibody assessment and its correlation with vaccine efficacy, anti-Vi IgG and IgA antibody estimates are accepted measures of TCV immunogenicity [1]. Although no international standard exists, a potential seroprotective threshold was defined as a fourfold or greater rise in anti-Vi antibody titers from baseline, based on results from field trials in children in Bangladesh [8], Nepal [10], and Malawi [9] that show 79%–85% efficacy.

A single dose of TCV is highly immunogenic in Burkinabe children vaccinated at the 15-month visit, providing both robust anti-Vi IgA and IgG responses at 28 days and durable immunity for 30–35 months after vaccination. Sustained immunogenicity for over 2.5 years after vaccination at 15 months informs vaccination schedule planning in endemic areas and suggests a booster dose, if needed, can likely wait until school entry. Future studies should continue to assess the longevity of vaccine-induced responses and the potential need for booster dosing in target populations.

## Supplementary Material

piad058_suppl_Supplementary_Tables

## Data Availability

After publication, the authors will provide data that underlie the results reported, after de-identification (text, tables, figures), to researchers who provide a methodologically sound proposal with approved aims. Proposals should be directed to mlaurens@som.umaryland.edu; to gain access, data requestors will need to sign a data use agreement.

## Notes

*Financial support.* The work was supported in part by a grant from the Bill & Melinda Gates Foundation [OPP1151153 to K.M.N] and we are grateful for their support.

***Potential conflicts of interest.*** S.D., J.K.T., T.G., L.P.J., K.M.N., and M.B.L. receive salary from the Typhoid Vaccine Acceleration Consortium (TyVAC) grant from the Bill & Melinda Gates Foundation (the Foundation). A.O., A.D., I.N., N.B., J.M.K., A.B.T., and S.B.S. receive funding from the University of Maryland, Baltimore. M.P. receives funding from NIH, NIAID, CDC, DARPA, Wellcome Trust, Vaxcyte, and the Foundation and J.O. and I.M. works on a sub-set of these grants. Y.L. receives funding from the following grants: R01NS122855 (National Institutes of Health, NIH), U54CK000615-01 (United States Centers for Disease Control and Prevention, CDC), 1R18 HS027750-01 (Agency for Healthcare Research and Quality), R03 AG067927 (NIH), U01 AI143493 (NIH), U01 AI148054 (NIH), U01 AI148081 (NIH), NCT04078022 (Institut Pasteur), HHS-NIH-NIAID-BAA2018 (NIH), R01 HD093946-01 (NIH), 2R01-DA026868-06A1 (NIH), 2R01-AA014988-12A1 (NIH), R01 AA014988-S1 (NIH), R01 DK109323 (NIH), R01-NR016269 (NIH), Geneva Foundation (National Institute of Allergy and Infectious Diseases [NIAID] prime), R01-HD075936 (NIH), and BAA FY2018-OADS-01 (CDC). In addition to TyVAC, M.B.L receives funding from UM1AI148689 (NIH), 1U01AI155300-01A1 (NIH), HHSN272201300022I (NIH), UM1AI148689 (NIH), HHSN272201300022I (NIH).

A.O., A.D., I.N., N.B., J.M.K., A.B.T., S.D. Y.L., I.M., J.O., J.K.T., T.G., M.P., L.P.J., S.B.S., and M.B.K. have no competing interests to declare. KMN is on the World Health Organization Strategic Advisory Group of Experts on Immunization (SAGE).
