# Supplementary material for: Durable Anti-Vi IgG and IgA Antibody Responses in 15-Month-Old Children Vaccinated With Typhoid Conjugate Vaccine in Burkina Faso
Source: J Pediatric Infect Dis Soc. 2023 Aug 17;12(9):513–8. doi: 10.1093/jpids/piad058 (PMC10533206; doi:10.1093/jpids/piad058)
Supplement: piad058_suppl_Supplementary_Tables [file piad058_suppl_supplementary_tables.docx]

**Supplementary Information**

1. Table A1: Baseline Demographics of Participants Returning or Lost to follow up at 30-35 Months Visit
2. Table A2: Anti-Vi IgG antibody geometric mean titers before vaccination (day 0), 28 days and 30-35 months after vaccination, by sex
3. Table A3: Anti-Vi IgA antibody geometric mean titers before vaccination (day 0), 28 days and 30-35 months after vaccination, by sex
4. Study protocol

**Table A1: Baseline demographics of participants returning or lost to follow up at 30-35 months visit**

|  | **Returned at 30-35 months** | | | **Did not return at 30-35 months** | | |  |
| --- | --- | --- | --- | --- | --- | --- | --- |
|  | **Group 1:**  **TCV +IPV**  **(delayed MCV-A)** | **Group 2:**  **TCV +MCV-A** | **Group 3:**  **MCV-A +IPV** | **Group 1:**  **TCV +IPV**  **(delayed MCV-A)** | **Group 2:**  **TCV +MCV-A** | **Group 3:**  **MCV-A +IPV** | **p-value^a^** |
| **Vaccinated** | 38 | 33 | 44 | 11 | 17 | 7 |  |
| **Sex** |  |  |  |  |  |  | 0.69**^a^** |
| **Female** | 16 (42.1%) | 15 (45.5%) | 26 (59.1%) | 3 (27.3%) | 8 (47.1%) | 5 (71.4%) |  |
| **Male** | 22 (57.9%) | 18 (54.6%) | 18 (40.9%) | 8 (72.7%) | 9 (52.9%) | 2 (28.6%) |  |
| **Age (months)** | 16.3 ±1.6 | 16.1 ±1.7 | 15.5 ±0.8 | 17.1 ±1.9 | 16.2±1.8 | 16.6±2.6 | 0.02^b^ |
| **Height** | 75.2 ±3.9 | 75.8 ±2.8 | 75.4 ±4.4 | 77.2 ±3.5 | 75.6 ±4.0 | 75.5 ±4.3 | 0.37^c^ |
| **Weight** | 9.3 ±1.2 | 9.5 ±0.9 | 9.5 ±1.2 | 9.5 ±1.1 | 9.6 ±1.5 | 9.4 ±1.6 | 0.72^c^ |
| **^a^** Chi-square test.  **^b^** Mann-Whitney U test.  **^c^** Two-sample t-test with unequal variances | | | | | | | |

**Table A2: Anti-Vi IgG antibody geometric mean titers before vaccination (day 0), 28 days and 30-35 months after vaccination, by sex**

|  |  | Male | | Female | |  |
| --- | --- | --- | --- | --- | --- | --- |
|  |  | **n** | **GMT (95% CI)** | **n** | **GMT (95% CI)** | **p-value^a^** |
| Group 1: TCV + IPV  (delayed MCV-A) | Day 0 | 30 | 4.3 (3.6-5.2) | 19 | 6.1 (3.8- 9.8) | 0.267 |
|  | Day 28 | 28 | 2307.4 (1058.3-5030.7) | 19 | 3574.8 (1613.4-7920.9) | 0.729 |
|  | Month 30-35 | 22 | 73.5 (47.1- 114.6) | 16 | 96.9 (65.3- 143.7) | 0.686 |
| Group 2: TCV + MCV-A | Day0 | 27 | 5.0 (3.5-7.1) | 23 | 4.3 (3.5-5.4) | 0.518 |
|  | Day 28 | 27 | 4051.4 (3022.8-5429.9) | 23 | 3340.5 (1764.2-6325.2) | 0.658 |
|  | Month 30-35 | 18 | 74.8 (43.4- 128.9) | 15 | 76.7 (55.9- 105.3) | 0.918 |
| Group 3: MCV-A + IPV | Day 0 | 20 | 4.5 (3.1- 6.7) | 31 | 5.0 (3.8- 6.8) | 0.618 |
|  | Day 28 | 20 | 5.6 (3.7- 8.6) | 31 | 5.1 (3.7- 7.0) | 0.902 |
|  | Month 30-35 | 18 | 6.2 (4.0- 9.6) | 26 | 5.4 (4.1- 7.1) | 0.733 |
| n=number of participants. GMT=geometric mean titer. CI=confidence interval. | | | | | | |
| ^a^Comparison of GMT between males and females using two sample t-test on log_10_ transformed data. | | | | | | |

**Table A3: Anti-Vi IgA antibody geometric mean titers before vaccination (day 0), 28 days and 30-35 months after vaccination, by sex**

|  |  | Male | | Female | |  |
| --- | --- | --- | --- | --- | --- | --- |
|  |  | **n** | **GMT (95% CI)** | **n** | **GMT (95% CI)** | **p-value^a^** |
| Group 1: TCV + IPV (delayed MCV-A) | Day 28 | 28 | 41.1 (26.2-64.5) | 19 | 52.8 (36.8-76.0) | 0.703 |
|  | Month 30-35 | 22 | 6.2 (4.1- 9.4) | 16 | 5.0 (3.0- 8.5) | 0.528 |
| Group 2: TCV + MCV-A | Day 28 | 27 | 39.6 (29.5-53.1) | 23 | 34.8 (23.7-51.0) | 0.589 |
|  | Month 30-35 | 18 | 5.0 (3.2- 7.6) | 15 | 4.1 (2.9- 5.7) | 0.524 |
| Group 3: MCV-A + IPV | Day 28 | 20 | 1.6 (1.6- 1.6) | 31 | 1.7 (1.5-1.9) | 0.799 |
|  | Month 30-35 | 18 | 1.7 (1.3- 2.2) | 26 | 1.7 (1.5- 2.1) | 0.945 |
| n=number of participants. GMT=geometric mean titer. CI=confidence interval. | | | | | | |
| ^a^Comparison of GMT between males and females using two sample t-test on log_10_ transformed data. | | | | | | |
